# Supplementary material for: The Extreme Environment Microbiome Catalog (EEMC): a global resource for microbial diversity and antimicrobial discovery
Source: Nat Commun. 2026 Apr 2;17:4791. doi: 10.1038/s41467-026-71145-0 (PMC13219616; doi:10.1038/s41467-026-71145-0)
Supplement: Supplementary file 1 — Supplementary Information [file 41467_2026_71145_MOESM1_ESM.pdf]

Supplementary information

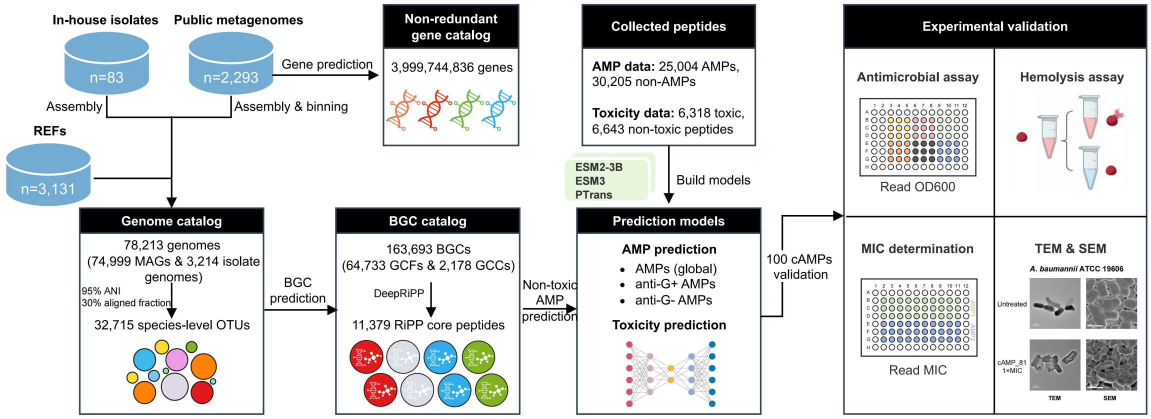

Supplementary Fig. 1. A schematic representation of study workflow. First, quality-controlled, high-throughput DNA sequencing reads from 2,293 extreme environment samples were individually assembled into metagenomic contigs and bins, resulting in 1) the establishment of the non-redundant gene catalog with 3,999,744,836 genes, and 2) 74,999 MAGs meeting the MIMAG criteria of contamination < 10% and completeness $\geq$ 50%. These MAGs were complemented with 83 genomes from in-house cultured isolates and 3,131 public REFs from NCBI RefSeq Genome database. The combined set of 78,213 genomes was clustered into 32,715 species-level OTUs, along with taxonomic annotation and prediction of BGCs and RiPP core peptides. Further, pre-trained pLLMs were used to build AMP models and non-toxic peptides models for predicting non-toxic AMPs from microbial RiPP core peptides. cAMPs were selected and subjected to in vitro validation, including antimicrobial assays, hemolysis assays, MIC determination, TEM and SEM. This figure was created with BioRender.com (https://BioRender.com/0i8r6by). MIMAG Minimum Information about a Metagenome-Assembled Genome, MAG metagenome-assembled genome, REF reference isolate genome, OTU operational taxonomic unit, ANI average nucleotide identity, BGC biosynthetic gene cluster, RiPP ribosomally synthesized and posttranslationally modified peptide, AMP antimicrobial peptide, cAMP candidate non-toxic antimicrobial peptide, G+ Gram-positive bacteria, G- Gram negative bacteria, MIC minimum inhibitory concentration, TEM transmission electron microscope, SEM scanning electron microscope.

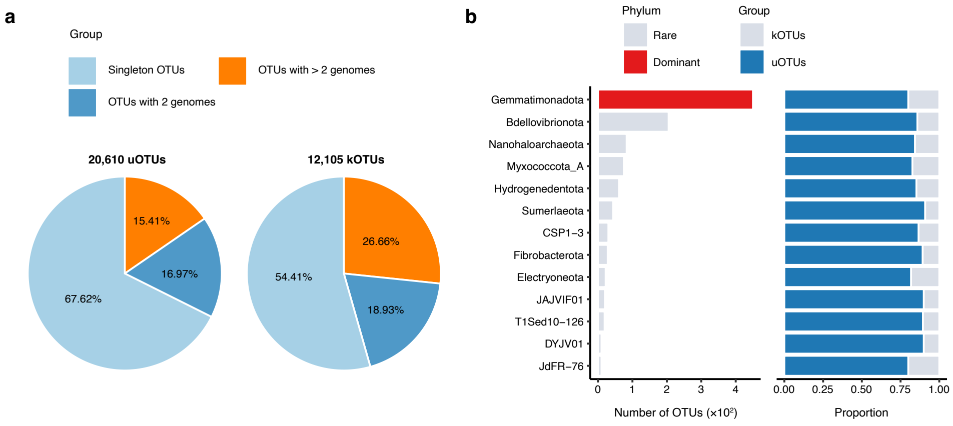

Supplementary Fig. 2. **Singleton rates and taxonomic patterns of uOTUs and kOTUs. a,** The proportion of singleton genomes within uOTUs and kOTUs. **b,** The OTU counts (left panel) and composition (right panel) in phyla with ≥ 10 OTUs and ≥ 80% uOTUs. uOTU unclassified OTU, kOTU known OTU.

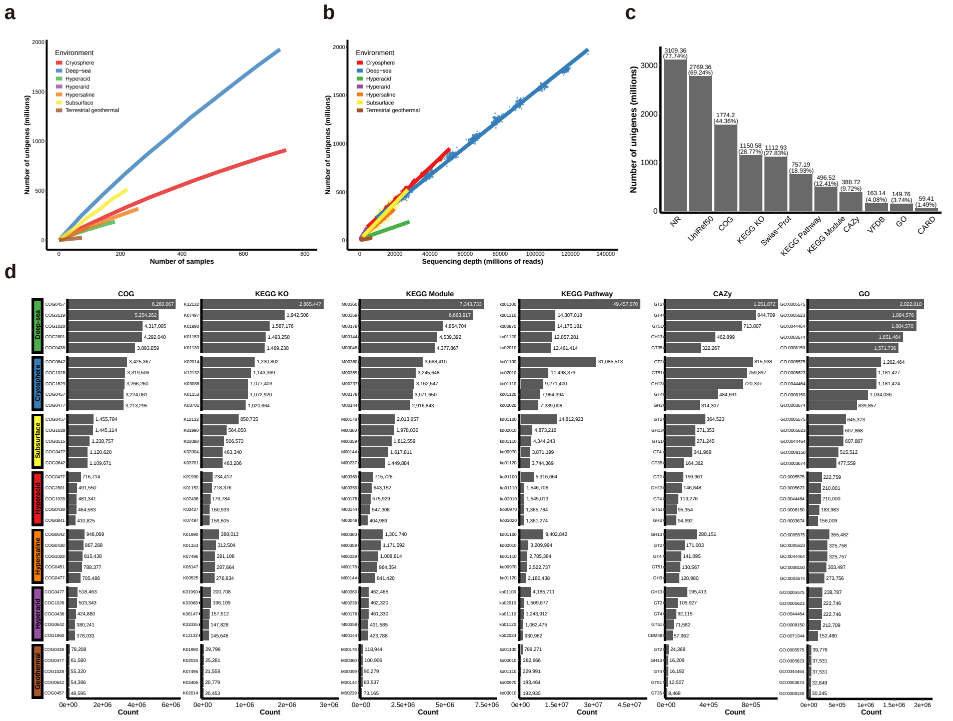

Supplementary Fig. 3. **Diversity and functional annotation of the unigenes across environments. a,** Unigene accumulation curves for each extreme environment, with line colors representing the corresponding environment types. **b,** Correlation between the number of unigenes and sequencing depth. Dot indicates each sample, and line colors represent the extreme environment type of each sample. **c,** Number and proportion of unigenes annotated in different databases. **d,** Top five annotated functions identified in each extreme environment based on different databases.

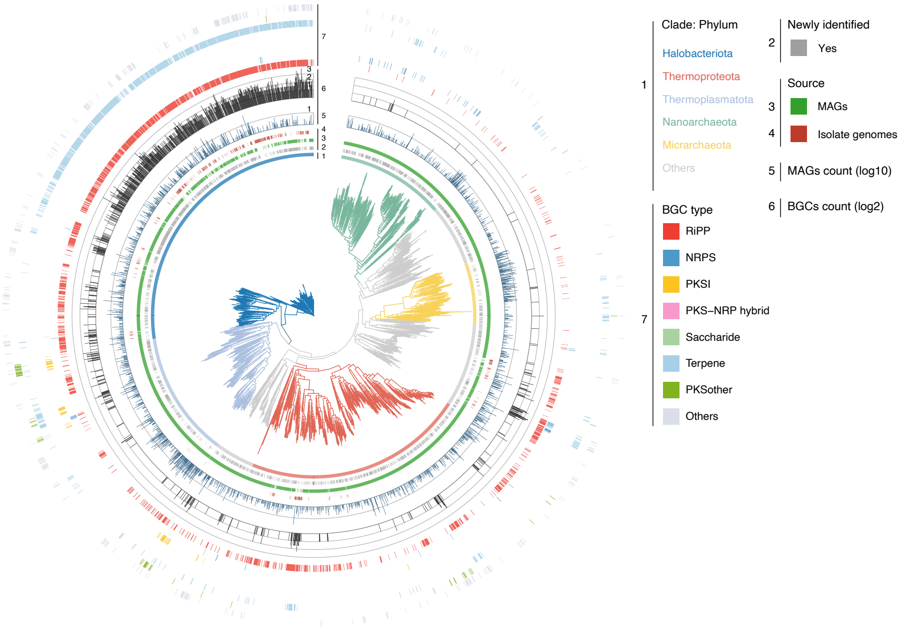

Supplementary Fig. 4. An archaeal phylogenetic tree. The tree was built for 32,715 representative OTUs based on a concatenated alignment of 122 universally distributed archaeal single-copy genes. The colors of the branches and the nearest outer layer (1) indicate the corresponding phyla. The other outer layers indicate, for each species, the novelty compared with the GTDB r220 database (2), the source of the genomes (3, 4), the genome count (5), the highest BGC count among its genomes (6), and the presence of each BGC type among its genomes (7).

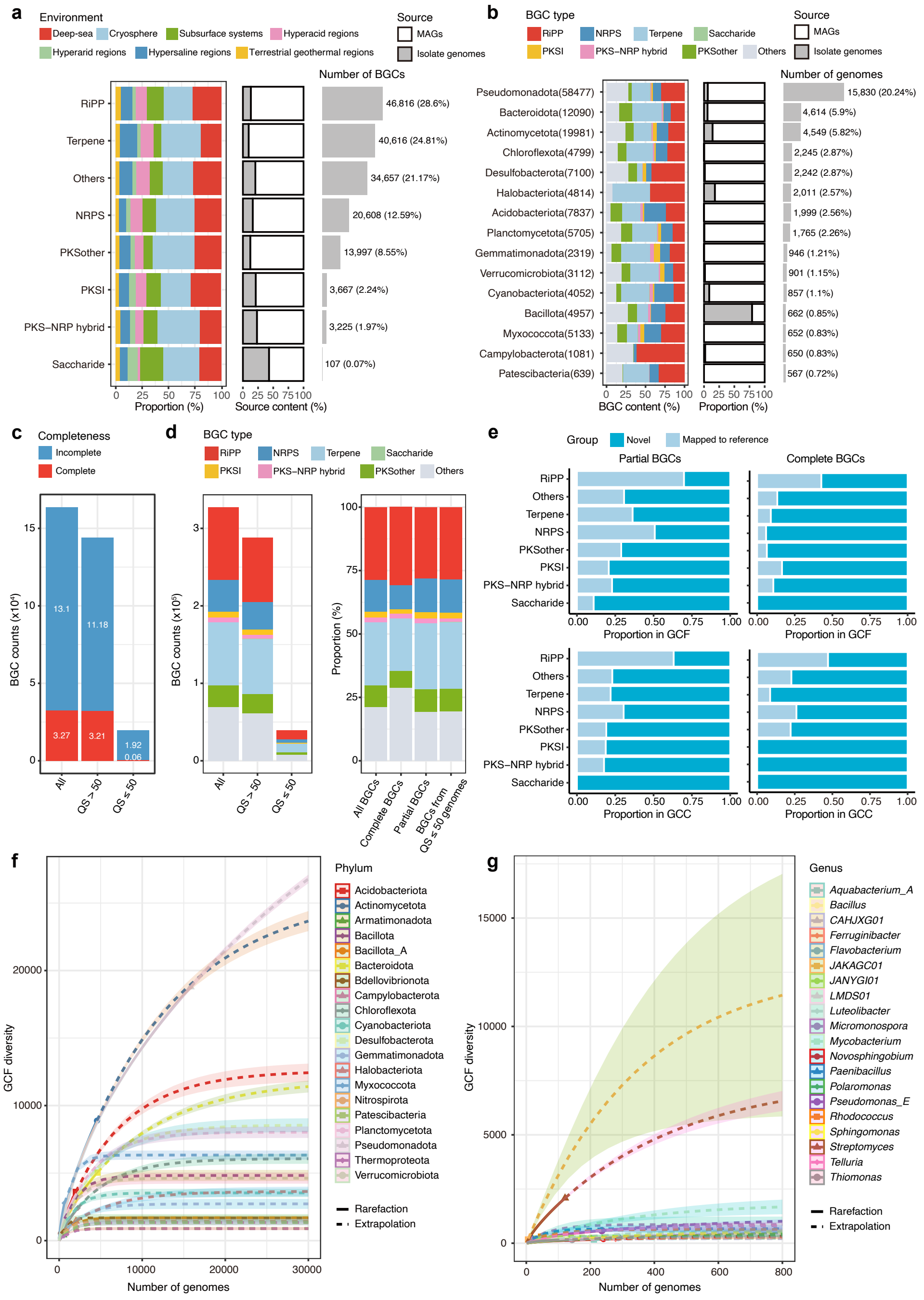

Supplementary Fig. 5. BGC distribution and diversity. a, The left panel shows the habitat (source) of each BGC type. The middle panel shows the percentage of BGCs identified in MAGs and isolate genomes. The right panel shows the count of each BGC type. **b,** The left panel shows the content of each BGC type across the dominant phyla. The middle panel shows the percentage of genomes identified in MAGs and isolate genomes. The right panel shows the count of genomes of each phylum. **c,** Completeness of BGCs derived from the full genome and QS-filtered genome sets. **d,** Counts of BGC classes across the full and QS-stratified genome sets (left panel), and relative proportions of BGC classes in the full, complete, partial, and lower-QS genome-derived BGC datasets (right panel). **e,** Novelty of GCCs and GCFs in partial and complete BGC sets. **f-g,** Rarefaction curves of the top 20 phyla (**f**) and top 20 genera (**g**) with the largest numbers of predicted GCFs. Source data are provided in this paper. QS quality score, GCF gene cluster family, GCC gene cluster clan.

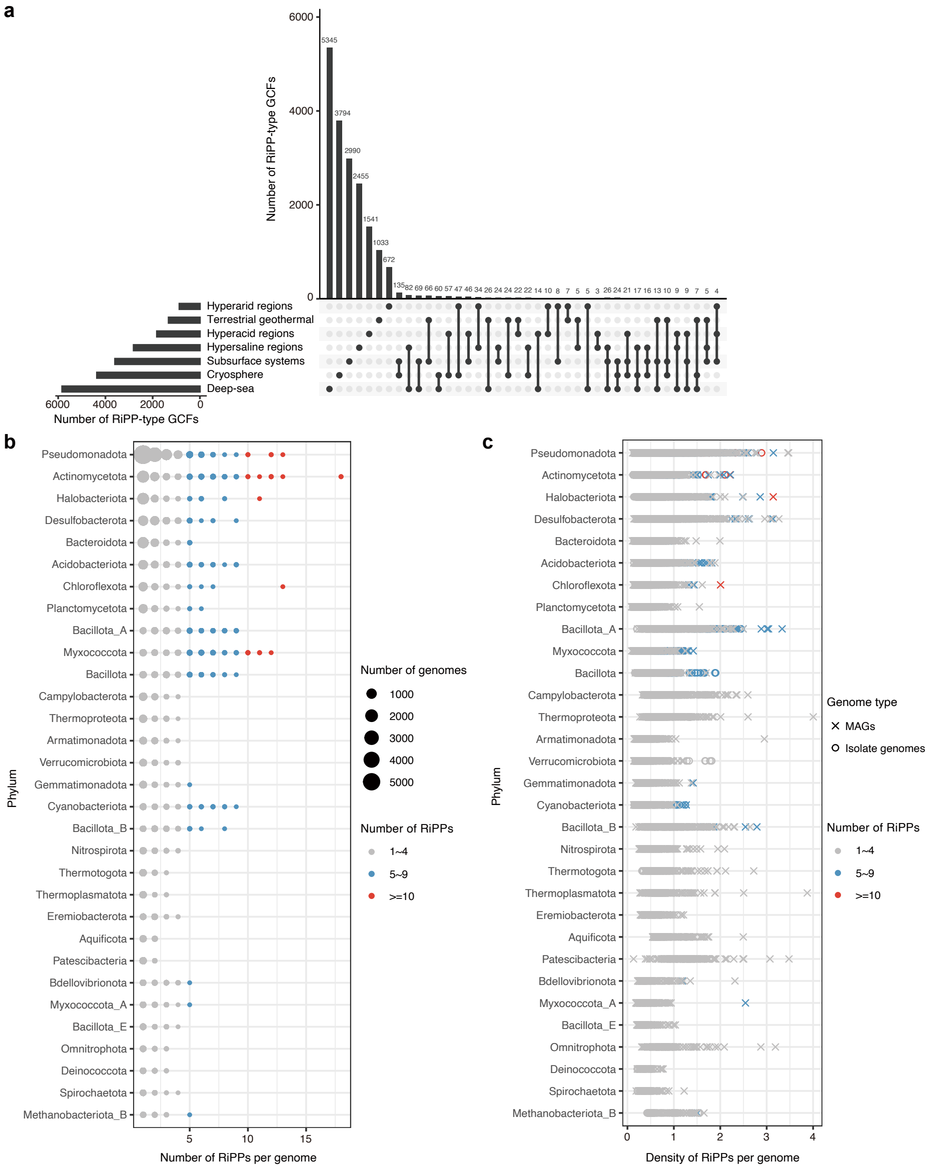

Supplementary Fig. 6. Diversity and distribution of RiPPs across extreme environments and dominant phyla. a, Environmental distributions of GCFs clustered from RiPP BGCs. **b,** Counts of RiPPs across the dominant phyla with at least 100 RiPP BGCs. The dot size indicates the number of genomes encoding RiPPs. The dot color indicates the group of RiPP counts. **c,** Density of RiPPs across the dominant phyla with at least 100 RiPP BGCs. The marker shape indicates the genome type (MAG or isolate genome). The dot color indicates the group of RiPP counts.

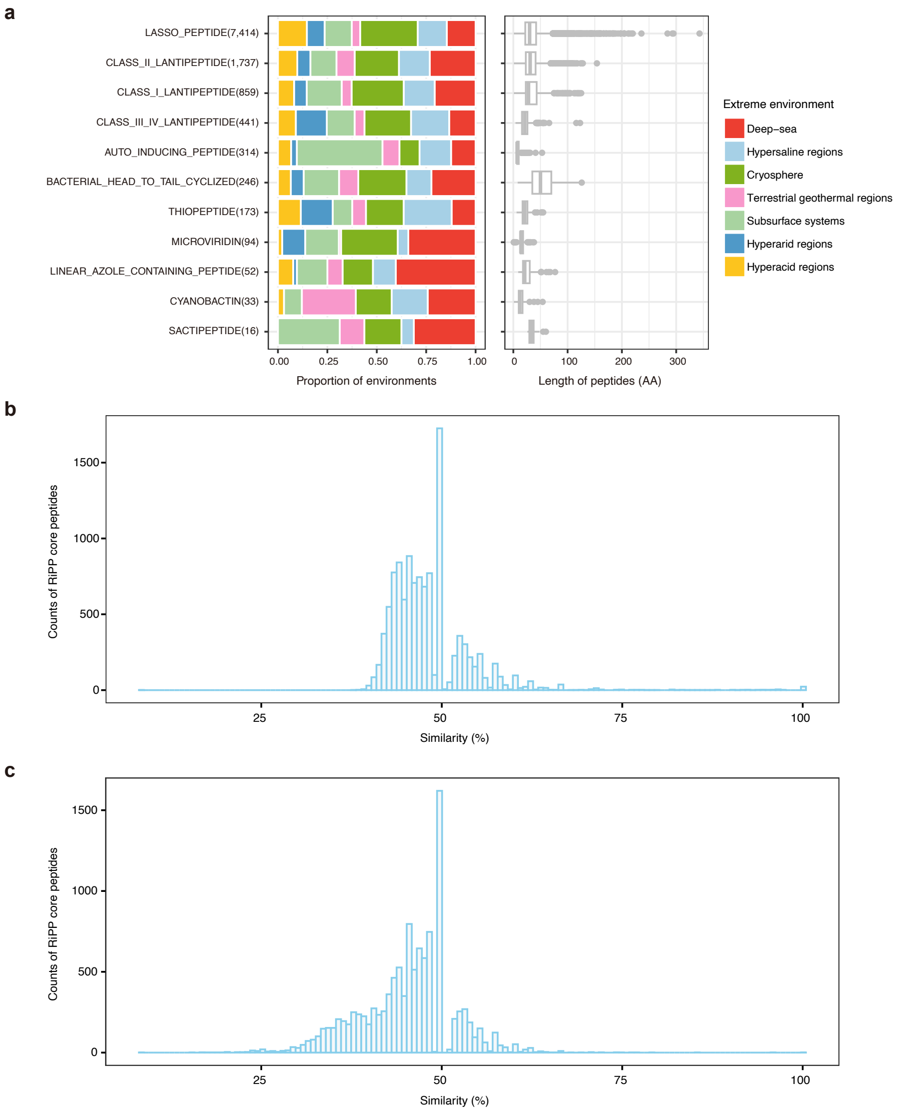

Supplementary Fig. 7. Distribution and lengths of 11,379 RiPP core peptides in extreme environments and their similarity with peptides of the training datasets. a, The left panel shows the distribution of the 11,379 RiPP core peptides across different extreme environments. The counts of each RiPP type are indicated on the y-axis. The right panel shows the distribution of lengths of the core peptides. **b-c,** Distribution of highest similarity between 11,379 RiPP core peptides to peptides of the global AMP training dataset (**b**) and toxicity training dataset (**c**).

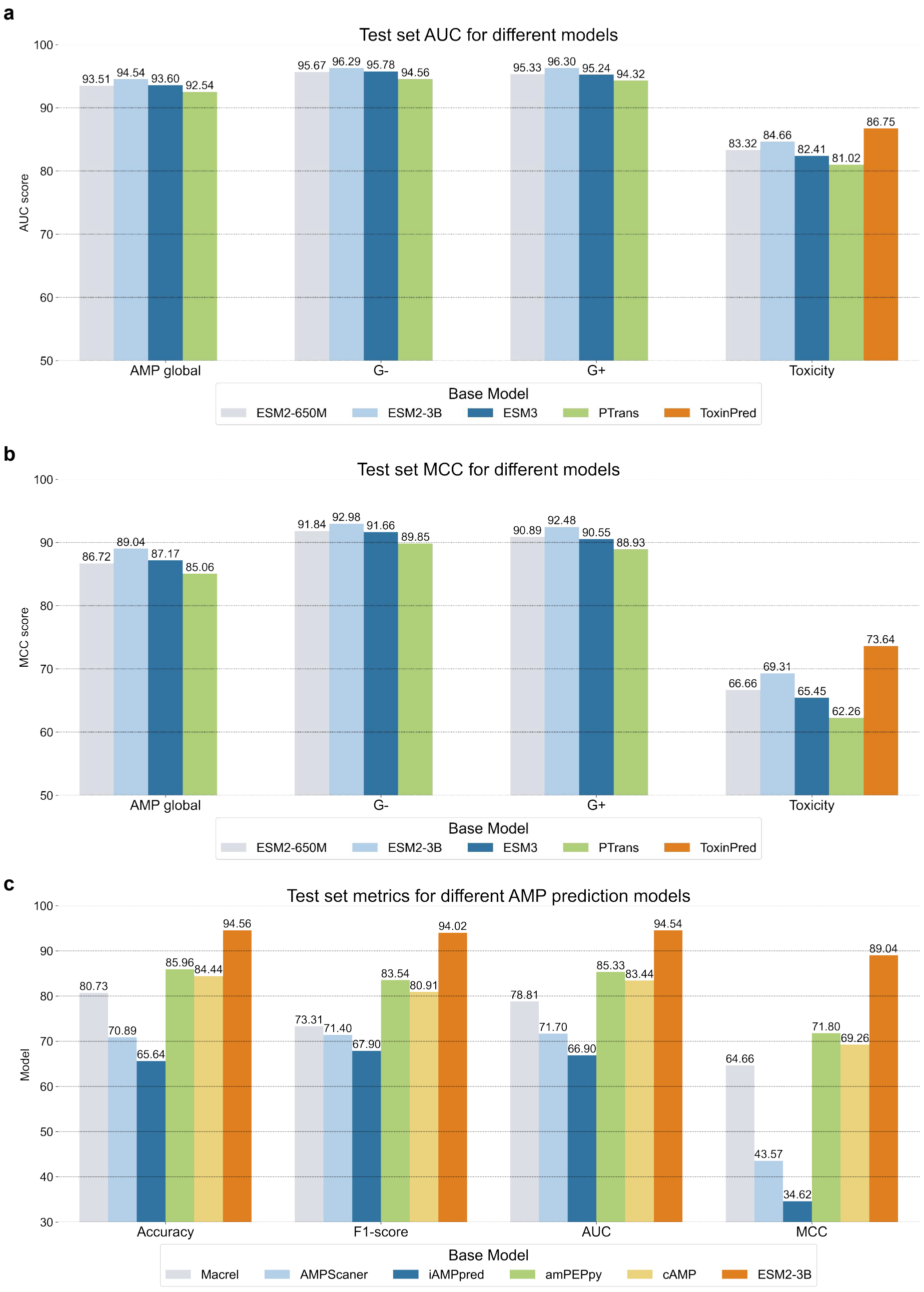

Supplementary Fig. 8. Performance of different models on test datasets. a-b, AUC (**a**) and MCC (**b**) of the ESM2-650M-, ESM2-3B-, ESM3-, PTrans-based models and ToxinPred on test datasets with binary labels (Global AMP, G+, G- or toxicity). **c,** Classification metrics of different AMP prediction tools, including Macrel, AMPScanner, iAMPpred, amPEPpy, c_AMP model, and our model trained with ESM2-3B, on the global AMP test dataset (5,521 sequences). AUC area under the receiver-operating characteristic curve, MCC Matthews correlation coefficient. G+ Gram-positive bacteria, G- Gram-negative bacteria.

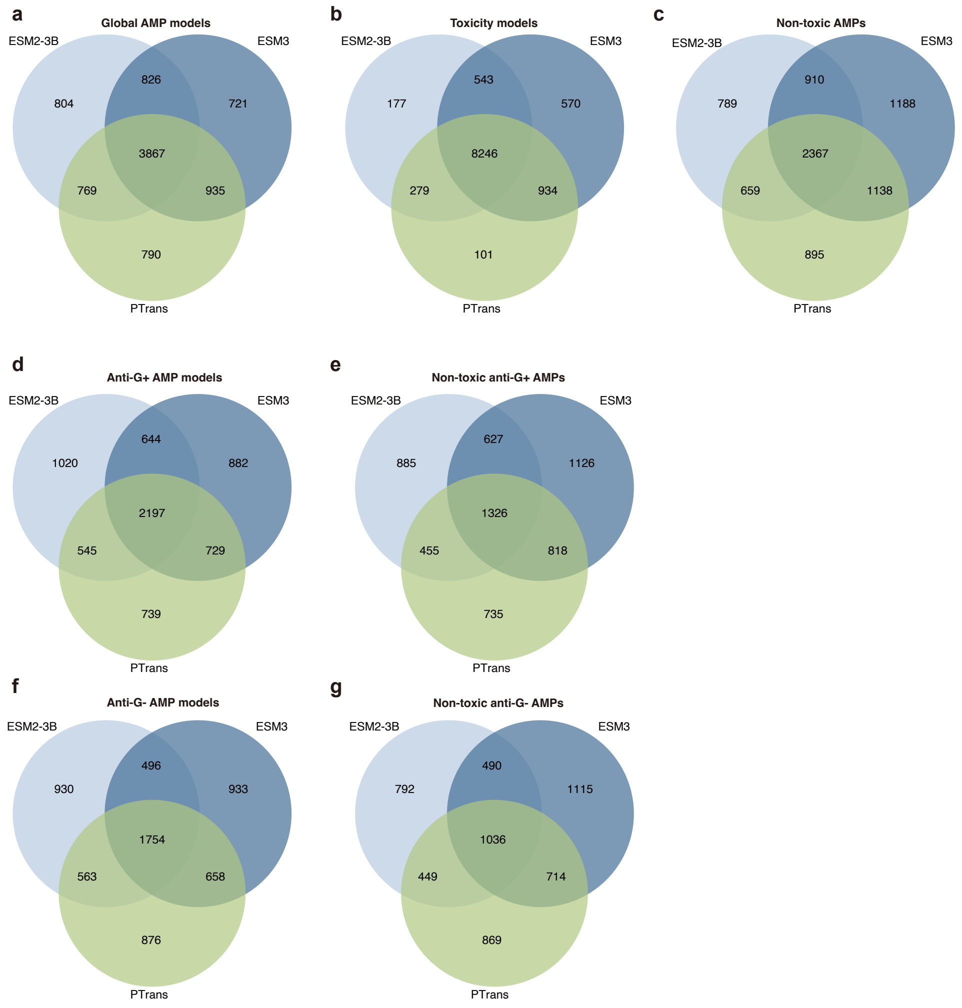

Supplementary Fig. 9. Venn diagrams show the overlap of the predictions by each model. Venn diagrams showing the overlap of AMPs against microbes(**a**), Gram-positive bacteria (**d**) and Gram-negative bacteria (**f**) identified by the ESM2-3B, ESM3 and PTrans models. Venn diagrams showing the overlap of non-toxic peptides identified by the ESM2-3B, ESM3 and PTrans models (**b**). Venn diagrams show the overlap of non-toxic AMPs against microbes (**c**), Gram-positive bacteria (**e**) and Gram-negative bacteria (**g**) identified by the ESM2-3B-, ESM3- and PTrans-based models.

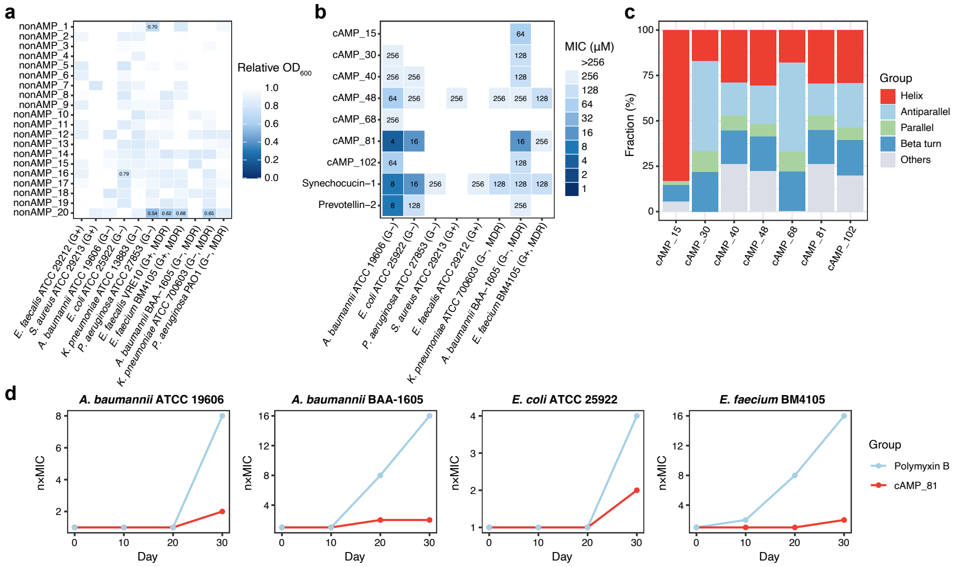

Supplementary Fig. 10. Antimicrobial activity of cAMPs and non-AMPs, resistance evolution, and secondary structure characterization. a, The heatmap shows the relative OD_{600} values of the cells grown in medium supplemented with candidate non-AMPs at a concentration of 60 μM, normalized to the OD_{600} of the cells grown in unsupplemented medium. Only the relative OD_{600} below 80% is labeled. **b,** Determination of MICs of the seven potent cAMPs and two published AMPs (prevotellin-2 and synechocucin-1) against eight commonly studied pathogens. **c,** The CDNN results of the CD assays for the 7 selected cAMPs. **d,** Longitudinal resistance assays for cAMP_81 and polymyxin B against *A. baumannii* ATCC 19606, *E. coli* ATCC 25922, MDR *E. faecium* BM4105, and MDR *A. baumannii* BAA-1605. The y-axis shows the fold change of MIC relative to the initial (day 0) MIC. The x-axis shows the passage points at which MIC was measured (days 0, 10, 20, and 30). CD circular dichroism, MDR multi-drug resistant.

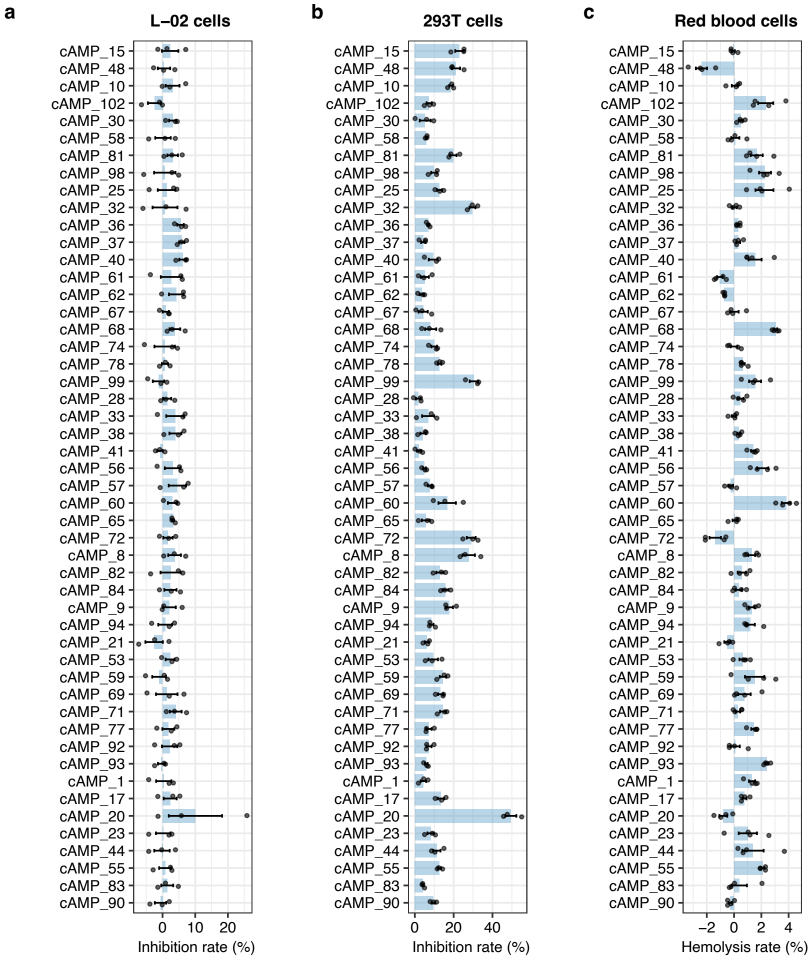

Supplementary Fig. 11. The results of the cytotoxicity tests on the L-2 cells (**a**) and 293T cells (**b**), and hemolysis assays (**c**) for the 50 selected cAMPs. The inhibition rates (n=3 biological replicates) and hemolysis rates (n=4 biological replicates) are presented as the mean values ± s.e.m. Source data are provided in this paper.

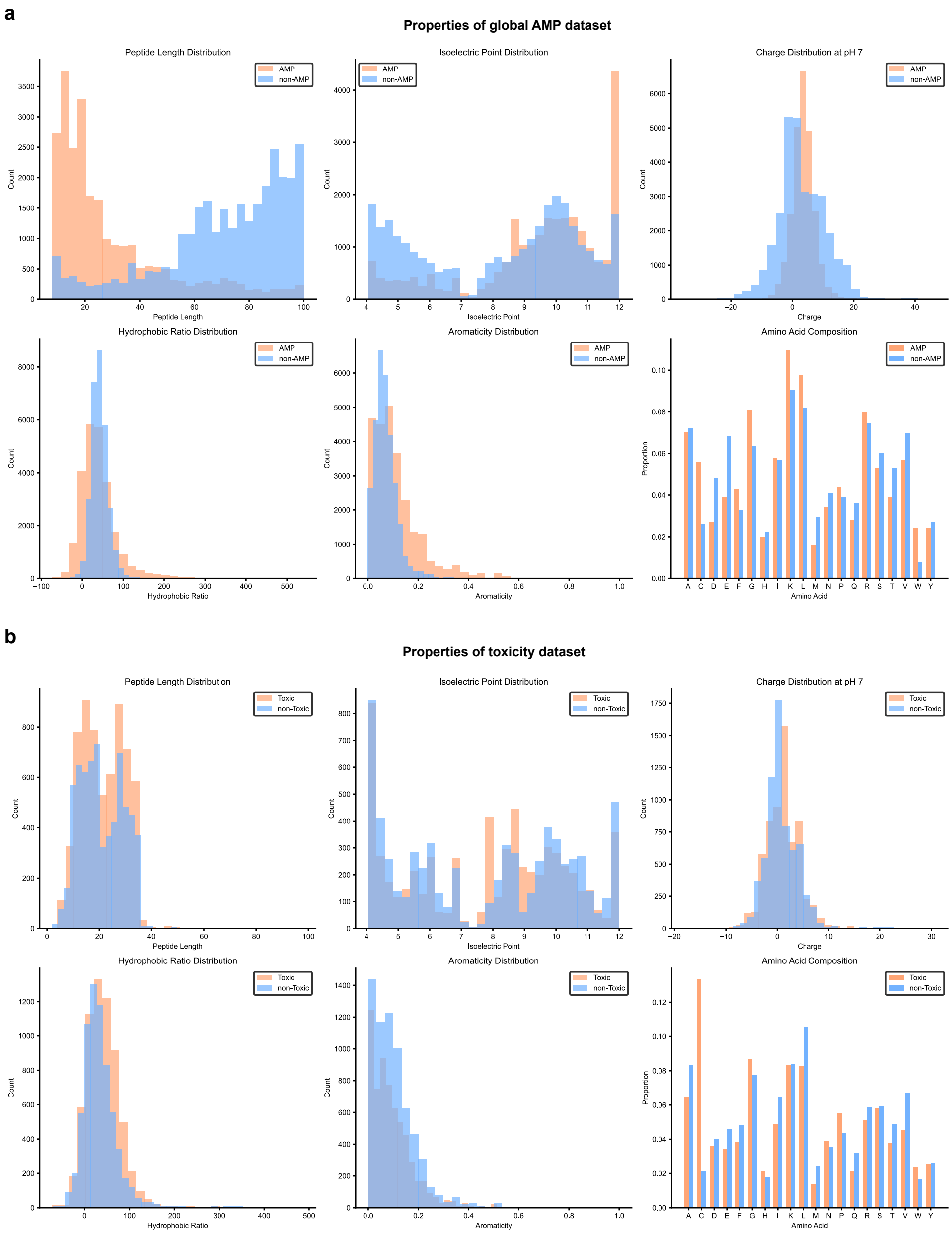

Supplementary Fig. 12. Distribution of peptide properties of the global AMP dataset (**a**) and toxic peptides dataset (**b**). The x-axis indicates the property type, and the y-axis indicates the number of peptides.

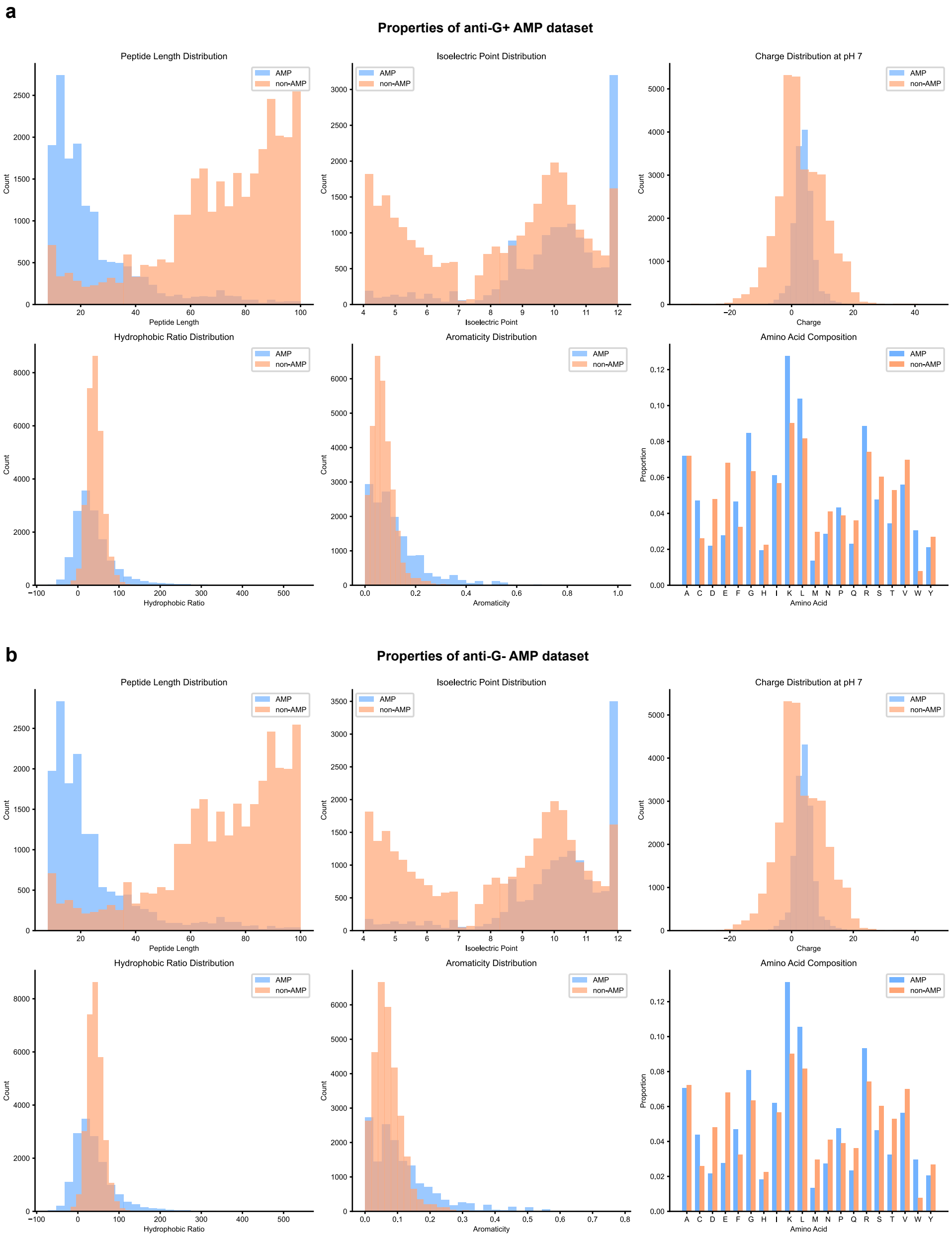

Supplementary Fig. 13. Distribution of peptide properties of the anti-G+ AMP dataset (**a**) and anti-G- AMP dataset (**b**). The x-axis indicates the property type, and the y-axis indicates the number of peptides. G+ Gram-positive bacteria, G- Gram-negative bacteria.
