# Supplementary material for: The Extreme Environment Microbiome Catalog (EEMC): a global resource for microbial diversity and antimicrobial discovery
Source: Nat Commun. 2026 Apr 2;17:4791. doi: 10.1038/s41467-026-71145-0 (PMC13219616; doi:10.1038/s41467-026-71145-0)
Supplement: Supplementary file 2 — Description of Additional Supplementary Files [file 41467_2026_71145_MOESM2_ESM.pdf]

Description of Additional Supplementary Files

Supplementary Data 1. The information of metagenomes collected from public databases.

Supplementary Data 2. The characteristics of the 78,213 genomes of the EEMC.

Supplementary Data 3. The comparison between the representative EEMC OTUs to the representative genomes from TARA, GEM and GOMC catalogs. The genomes with ANI ≥ 0.95 and coverage ≥ 0.3 were clustered into one secondary cluster. The secondary clusters containing both EEMC OTUs and TARA, GEM or GOMC catalogs were shown.

Supplementary Data 4. Functional annotation of the gene catalog across environments. The top 20 annotations of each database are displayed.

Supplementary Data 5. Alignment and annotation of the core unigene set shared among all environments against the NCBI NR database.

Supplementary Data 6. The characteristics of the 163,693 biosynthetic gene clusters.

Supplementary Data 7. The counts of biosynthetic gene clusters in top15 phyla.

Supplementary Data 8. The environmental distribution of the biosynthetic gene clusters.

Supplementary Data 9. The novelty of the gene cluster families (GCFs) and gene cluster clans (GCCs) of the biosynthetic gene clusters.

Supplementary Data 10. The counts of GCFs at the level of phylum.

Supplementary Data 11. The counts of the RiPP-type GCFs at the level of phylum.

Supplementary Data 12. The counts and density of the RiPPs in the dominant phyla with at least 100 RiPP-type BGCs.

Supplementary Data 13. Databases utilized for AMPs and their respective counts.

Supplementary Data 14. The performance of MAI models and published models.

Supplementary Data 15. The information and predicted results of 11,379 RiPP corepeptides.

Supplementary Data 16. The detailed information of 100 cAMPs and 20 non-AMPs.

Supplementary Data 17. The relative OD600 of 100 cAMPs and 20 candidate non-AMPs against eleven pathogenic strains.

Supplementary Data 18. The MIC values (μM) of 20 cAMPs and published AMPs against eight strains.

Supplementary Data 19. The inhibition rates and hemolysis rates of 50 cAMPs in L-02, 293T and red blood cells.

Supplementary Data 20. The CC50 values (μM) of 7 cAMPs in L-02 cells.

Supplementary Data 21. Fold changes (relative to Day0) of MIC values for cAMP_81 and polymyxin B against four pathogenic strains during longitudinal resistance assays.

Supplementary Data 22. The regular expression keywords used to filter isolate genomes derived from extreme environments.
