# Supplementary material for: The Extreme Environment Microbiome Catalog (EEMC): a global resource for microbial diversity and antimicrobial discovery
Source: Nat Commun. 2026 Apr 2;17:4791. doi: 10.1038/s41467-026-71145-0 (PMC13219616; doi:10.1038/s41467-026-71145-0)
Supplement: Supplementary file 6 — Reporting Summary [file 41467_2026_71145_MOESM6_ESM.pdf]

nature portfolio

Corresponding author(s): Haixin Chen

Last updated by author(s): Mar 12, 2026

# Reporting Summary

Nature Portfolio wishes to improve the reproducibility of the work that we publish. This form provides structure for consistency and transparency in reporting. For further information on Nature Portfolio policies, see our Editorial Policies and the Editorial Policy Checklist.

## Statistics

For all statistical analyses, confirm that the following items are present in the figure legend, table legend, main text, or Methods section.

| n/a | Confirmed | |
|---|---|---|
| ☐ | ☒ | The exact sample size (*n*) for each experimental group/condition, given as a discrete number and unit of measurement |
| ☐ | ☒ | A statement on whether measurements were taken from distinct samples or whether the same sample was measured repeatedly |
| ☒ | ☐ | The statistical test(s) used AND whether they are one- or two-sided<br>*Only common tests should be described solely by name; describe more complex techniques in the Methods section.* |
| ☒ | ☐ | A description of all covariates tested |
| ☒ | ☐ | A description of any assumptions or corrections, such as tests of normality and adjustment for multiple comparisons |
| ☐ | ☒ | A full description of the statistical parameters including central tendency (e.g. means) or other basic estimates (e.g. regression coefficient) AND variation (e.g. standard deviation) or associated estimates of uncertainty (e.g. confidence intervals) |
| ☒ | ☐ | For null hypothesis testing, the test statistic (e.g. *F*, *t*, *r*) with confidence intervals, effect sizes, degrees of freedom and *P* value noted<br>*Give P values as exact values whenever suitable.* |
| ☒ | ☐ | For Bayesian analysis, information on the choice of priors and Markov chain Monte Carlo settings |
| ☒ | ☐ | For hierarchical and complex designs, identification of the appropriate level for tests and full reporting of outcomes |
| ☒ | ☐ | Estimates of effect sizes (e.g. Cohen's *d*, Pearson's *r*), indicating how they were calculated |

*Our web collection on statistics for biologists contains articles on many of the points above.*

## Software and code

Policy information about availability of computer code

| | |
|---|---|
| Data collection | We collected and reanalyzed metagenomic sequencing data from samples collected from extreme environments with available metadata published from January 2010 to February 2024. A total of 3,131 publicly available bacterial and archaeal isolate genomes derived from extreme environments were downloaded from the NCBI RefSeq genome database (up to August, 2024). In addition, we derived bacteria from cold seep sediment and generated in-house isolate genomes. Briefly, sediment samples were retrieved from Haima cold seep field, northern South China Sea. The sediment samples were resuspended in sterile seawater and mixed thoroughly. The samples were subsequently diluted using gradient dilution method, and the dilutions were spread on 2216E medium (Park Hope, Shandong, China) and Gauze's Synthetic Medium No.1 (Park Hope, Shandong, China). The plates were then incubated in an inverted position at 28 °C. After several days of culture, single colonies were selected and transferred to new 2216E plates for purification. The isolates were then inoculated into glycerol tubes for storage at -80 °C. The cultivated isolates were cultured in 2216E broth at 28 °C, and cells were harvested by centrifugation at 4,000 x g for 10 minutes at the mid-log phase. Genomic DNA was extracted from the cultures using a TIANamp Bacteria DNA Kit (Tiangen, Beijing, China) and then sequenced by BGI using the DNBSEQ-T7 platform with 150-bp paired-end reads. |
| Data analysis | Raw metagenomic sequencing reads were obtained using sratoolkit (version 3.0.0) and transformed to fastq format using fastq-dump (version 2.9.6). Reads with low sequencing quality and adapter sequences were removed using fastp with default parameters (version 0.12.0). Clean data of each metagenomic sample were assembled by MEGAHIT (version 1.2.9) with the parameters "--presets meta-sensitive". The assembled contigs were binned and refined using MetaWRAP (version 1.3.2) with default parameters, which integrated the binning results of metaBAT2 (version 2.12.1), MaxBin2 (version 2.2.7) , and CONCOCT (version 1.1.0). A total of 77,146 refined MAGs were further refined using MAGpurify (version 2.1.2) with default modules and the parameters "phylo-markers", "clade-markers", "tetra-freq", "gc-content", and "known-contam" to remove contaminated contigs originating from a different species than the dominant organism in the MAG. For sequencing data of isolates, Fastp (version 0.12.0) was used for quality control of the paired-end reads; specifically, adapter sequences and low-quality reads were removed. The genomes of the cultivated isolates were assembled using Unicycler (version 0.5.0) with default |

parameters, and only contigs >= 500 bp were retained. Unicycler functions as a SPAdes-optimiser when applied to short-read only sets. The completeness and contamination of each purified MAG and isolate genomes were evaluated using the module "lineage_wf" in CheckM (version 1.2.1). The presence of ribosomal RNAs (rRNAs) and transfer RNAs (tRNAs) was identified using Infernal (version 1.1.15) with the parameters "--cut_ga, --rfam" and models from the Rfam database (version 14.10). The 78,213 genomes were clustered into 32,715 representative species-level OTUs using the module "dereplicate" in dRep (version 3.4.3), on the basis of an AF threshold of 30% and a genome-wide ANI threshold of 95% with the parameters "-nc 0.3, -sa 0.95, -comp 0, -con 1000". Taxonomic annotation of 32,715 representative OTUs was performed using the module "classify_wf" in the Genome Database Taxonomy Toolkit (GTDB-Tk, version 2.4.0) with default parameters against the GTDB release R220. Gene ORFs were predicted from metagenomic contigs using Prodigal (version 2.6.3) with the parameters "-p meta". All ORFs were clustered into non-redundant gene clusters at a threshold of 95% ANI and 80% AF, using MMseqs2 (version 113e321) with the parameters "easy-linclust -e 0.001 --min-seq-id 0.95 -c 0.80". We used Prodigal with default parameters for ORF prediction on the MAGs and isolate genomes from the extreme environments. All ORFs were clustered into non-redundant gene clusters at a threshold of 95% ANI and 80% AF, using MMseqs2(version 113e321). A total of 163,693 BGCs were predicted and identified on contigs >=5 kb of the 78,213 genomes to reduce the risk of fragmentation, using antiSMASH (version 7.0.1) with the parameters "--minlength 5000". We further use python 3 and R language (version 4.2) for analyzing results and generating plots.

For manuscripts utilizing custom algorithms or software that are central to the research but not yet described in published literature, software must be made available to editors and reviewers. We strongly encourage code deposition in a community repository (e.g. GitHub). See the Nature Portfolio guidelines for submitting code & software for further information.

## Data

Policy information about availability of data

All manuscripts must include a data availability statement. This statement should provide the following information, where applicable:

- Accession codes, unique identifiers, or web links for publicly available datasets
- A description of any restrictions on data availability
- For clinical datasets or third party data, please ensure that the statement adheres to our policy

All 74,999 MAGs generated in this study, together with 83 in-house isolate genomes from deep-sea, the non-redundant gene sets from assembled contigs and genomes, and 163,693 BGCs, have been deposited in the China National GeneBank DataBase (CNGBdb) with accession number CNP0007106 (https://db.cngb.org/search/project/CNP0007106/). The accession IDs of publicly available bacterial and archaeal reference genomes from NCBI genome database are provided in Supplementary Data 2. The referenced representative genomes used in this study, including 113,104 from GTDB release R220, 22,732 from GEM, 24,195 from GOMC, and 957 from Tara Ocean, are available at https://gtdb.ecogenomic.org/, https://portal.nersc.gov/GEM/genomes/, https://db.cngb.org/maya/datasets/MDB0000002, and https://merenlab.org/data/tara-oceans-mags/, respectively. The 4,472 representative genomes from UHGG v2.0 used in this study are available at https://www.ebi.ac.uk/metagenomics/genome-catalogues/human-gut-v2-0-2. All additional data supporting the findings of this study are provided within the article and its Supplementary Information files. Source data are provided with this paper.

## Research involving human participants, their data, or biological material

Policy information about studies with human participants or human data. See also policy information about sex, gender (identity/presentation), and sexual orientation and race, ethnicity and racism.

| | |
|---|---|
| Reporting on sex and gender | Not applicable. |
| Reporting on race, ethnicity, or other socially relevant groupings | Not applicable. |
| Population characteristics | Not applicable. |
| Recruitment | Not applicable. |
| Ethics oversight | Not applicable. |

Note that full information on the approval of the study protocol must also be provided in the manuscript.

# Field-specific reporting

Please select the one below that is the best fit for your research. If you are not sure, read the appropriate sections before making your selection.

☐ Life sciences ☐ Behavioural & social sciences ☒ Ecological, evolutionary & environmental sciences

For a reference copy of the document with all sections, see nature.com/documents/nr-reporting-summary-flat.pdf

# Ecological, evolutionary & environmental sciences study design

All studies must disclose on these points even when the disclosure is negative.

| | |
|---|---|
| Study description | This study compiles, curates and analyzes 2,293 publicly available metagenomes and 3,214 isolate genomes from extreme environments worldwide, generating a unified catalog of microbial genomes, genes, biosynthetic gene clusters (BGCs), and predicted antimicrobial peptides. The work is primarily computational, with limited in vitro assays for candidate antimicrobial peptides. |
| Research sample | The research sample consists of 2,293 metagenomic datasets and 3,131 isolate genomes retrieved from public repositories. In addition, we derived bacteria from cold seep sediment and generated 83 in-house isolate genomes. The sediment samples were |

| | |
|---|---|
| | retrieved from Haima cold seep field, northern South China Sea in 2023. The sequencing and assembly of isolates are described in the Methods. |
| Sampling strategy | In-house cold seep samples for this study were sourced from field sampling conducted using a uniform sampling protocol. |
| Data collection | Puzi Jiang, Zhengjiao Liang and Feng wang first screened published studies in the NCBI PubMed database in 2024, by searching keywords related to various extreme environments and ecosystems. We cross-referenced the identified datasets and removed duplicates. We further checked and removed samples of rRNA gene amplicon sequences, metatranscriptomic sequences and host-associated sequences. A total of 2,293 metagenomic samples with more than 23 Tb sequencing data were downloaded from the NCBI and NGDC databases. A total of 3,131 publicly available bacterial and archaeal isolate genomes derived from extreme environments were downloaded from the NCBI RefSeq genome database. Specifically, we downloaded the available metadata of 350,267 genomes from the database using the NCBI Datasets (up to August, 2024), which is useful to download biological sequence data across all domains of life from NCBI. The high quality genomes (completeness ≥ 95% and contamination < 5%) were selected if their isolation_source or geo_loc_name matched the regular expression of any extreme environment (Supplementary Data 22). We further curated the dataset by manually removing genomes derived from humans or other species. |
| Timing and spatial scale | The metagenomes and isolate genomes span samples collected globally between 2010 and 2024. Spatial coverage includes cryosphere, hypersaline, hyperarid, deep-sea, geothermal, hyperacid and subsurface systems. The in-house sediment samples were retrieved from Haima cold seep field, northern South China Sea in August 2023. |
| Data exclusions | No raw datasets were excluded except those failing standard quality-control criteria (e.g., missing metadata, incomplete sequencing, or corrupted files). All exclusions are described in the Methods. No experimental data were excluded from the analyses. |
| Reproducibility | All computational analyses were performed using published tools with version-controlled workflows. Curated datasets and trained models have been made publicly available for reproducibility. All in vitro experiment were performed with at least three techinical replicates, and all attempts at replications were successful. |
| Randomization | Randomization was not applicable to the in-house cold seep samples. These samples were collected prior to the initiation of this study as part of field sampling activities and were not assigned to experimental groups. Randomization was not applicable to the in vitro experiments. |
| Blinding | No blinding was required because all measurements are not subject to investigator's bias or ambiguity |

Did the study involve field work? ☐ Yes ☒ No

# Reporting for specific materials, systems and methods

We require information from authors about some types of materials, experimental systems and methods used in many studies. Here, indicate whether each material, system or method listed is relevant to your study. If you are not sure if a list item applies to your research, read the appropriate section before selecting a response.

## Materials & experimental systems

| n/a | Involved in the study |
|---|---|
| ☒ | ☐ Antibodies |
| ☐ | ☒ Eukaryotic cell lines |
| ☒ | ☐ Palaeontology and archaeology |
| ☒ | ☐ Animals and other organisms |
| ☒ | ☐ Clinical data |
| ☒ | ☐ Dual use research of concern |
| ☒ | ☐ Plants |

## Methods

| n/a | Involved in the study |
|---|---|
| ☒ | ☐ ChIP-seq |
| ☒ | ☐ Flow cytometry |
| ☒ | ☐ MRI-based neuroimaging |

# Eukaryotic cell lines

Policy information about cell lines and Sex and Gender in Research

| | |
|---|---|
| Cell line source(s) | L-02 (human hepatocyte cell line) was obtained from BNCC (BeNa Culture Collection, Beijing, China).<br>HEK293T cells were purchased from Procell Life Science & Technology (Hubei, China). |
| Authentication | Both L-02 and 293T cell lines were provided by the suppliers with certificates of analysis. Cell line identity was verified by the vendors using short tandem repeat (STR) profiling prior to shipment. No additional in-house authentication was performed. |
| Mycoplasma contamination | L-02 and 293T cells were routinely tested for mycoplasma contamination using PCR-based assays and were confirmed to be mycoplasma-free at the time of experiments. |
| Commonly misidentified lines<br>(See ICLAC register) | L-02 (HL-7702) is listed in the ICLAC Register of Misidentified Cell Lines (ICLAC ID: 190616) due to historical reports of HeLa cross-contamination. The L-02 cells used in this study were obtained from BNCC (Beijing, China), which provides an STR-authenticated version of this line. According to the supplier, the deposited strain had been authenticated and showed no evidence of cross-contamination. We did not perform additional STR profiling in our laboratory. |

## Plants

| | |
|---|---|
| Seed stocks | Not applicable. |
| Novel plant genotypes | Not applicable. |
| Authentication | Not applicable. |
